# An Efficient Agent Based Data Management Method of NoSQL Environments for Health Care Applications

**DOI:** 10.3390/healthcare9030322

**Published:** 2021-03-13

**Authors:** Theodore Kotsilieris

**Affiliations:** Department of Business and Organizations Administration, University of the Peloponnese, 24100 Kalamata, Greece; t.kotsilieris@uop.gr; Tel.: +30-27210-45255

**Keywords:** mobile agents, NoSQL, healthcare informatics, remote health data monitoring

## Abstract

Background: As medical knowledge is continuously expanding and diversely located, Health Information Technology (HIT) applications are proposed as a good prospect for improving not only the efficiency and the effectiveness but also the quality of healthcare services delivery. The technologies expected to shape such innovative HIT architectures include: Mobile agents (Mas) and NoSQL technologies. Mobile agents provide an inherent way of tackling distributed problems of accessing heterogeneous and spatially diverse data sources. NoSQL technology gains ground for the development of scalable applications with non-static and open data schema from complex and diverse sources. Methods and Design: This paper conducts a twofold study: It attempts a literature review of the applications based on the mobile agent (MA) and NoSQL technologies for healthcare support services. Subsequently, a pilot system evaluates the NoSQL technology against the relational one within a distributed environment based on mobile agents for information retrieval. Its objective is to study the feasibility of developing systems that will employ ontological data representation and task implementation through mobile agents towards flexible and transparent health data monitoring. Results and Discussion: The articles studied focus on applying mobile agents for patient support and healthcare services provision thus as to make a positive contribution to the treatment of chronic diseases. In addition, attention is put on the design of platform neutral techniques for clinical data gathering and dissemination over NoSQL. The experimental environment was based on the Apache Jena Fuseki NoSQL server and the JAVA Agent DEvelopment Framework -JADE agent platform. The results reveal that the NoSQL implementation outperforms the standard relational one.

## 1. Introduction

As medical data and knowledge are continuously expanding and diversely located, this work will evaluate whether Mobile Agent Systems (MASs) are a promising solution taking into account that the implementation and adoption of health information systems is a slow process due to the participation of several stakeholders (e.g., physicians, nurses, managers, patients, etc.) who hold varying expertise and duties.

According to World Health Organization, the population of 60+ aged people is expected to double by 2050, reaching 22% from 12% currently [1]. An in-depth insight of specific countries statistics reveals that the problem of population aging will be sharper in developing countries. In these countries, the population aged 60+ is expected to double within 20 years or less, while developed countries face the same problem within a timeframe of 150 years. Additionally, the problem of population aging incurs a social problem: Due to the modern way of life, these people tend to live alone and apart from their descendants.

Health Information Technology (HIT) applications are proposed as a good prospect for improving not only the efficiency and the effectiveness but also the quality of healthcare services delivery. The operational benefits that can be expected from HITs are: Cost and medical error reduction, enhancement of the quality of life of patients, and the advancement of medical decision-making tools. Currently, the potential of HIT to transform the delivery of health care has gathered vast research efforts. Although multipurpose HIT systems have emerged, their adoption is limited by the lack of interoperability, data sharing, and big data gathering—analysis that will improve health care provision.

Some commonly asked questions that HITs attempt to provide convincing evidence include: How could a HIT alert a user that critical medical indicators fall within dangerous range? How could a system take care of a patient that suffered a heart attack during the night by calling for help? Answers will allow for millions of people to be relieved or rescued, life expectancy will increase, and the sense of security will allow for aging at home. Within this framework, this work studies the importance of mobile software agents in forming an open and integrated platform for delivering advanced data retrieval and processing services over NoSQL databases.

The rest of this paper is organized as follows: Section 2 attempts a literature review along with the most significant applications and studies of mobile agents for remote medical services. Section 3 describes the experimental environment and briefly discusses the results obtained. Section 4 concludes the paper.

## 2. Materials and Methods

### 2.1. Literature Review and Key Challenges

Although the adoption of a common architecture for HITs is premature, the technology evolution rate is encouraging towards the implementation of widespread ICT-enabled healthcare services. The integration of new technologies along with the incurred benefits towards the development of a real-time health monitoring system is a challenge elicited by the research community. More specifically, the technologies expected to shape such an innovative architecture include: Autonomous decision-making agents, IoT devices and sensors, reliable and inexpensive cloud services, data security, and machine learning techniques in health data analysis.

A software agent is an autonomous computer application able to perform measurements that are necessary to accomplish its decision-making role. It is able to act on behalf of a user and possesses unique properties such as: (i) Multi-goals software entity, (ii) context awareness, and the agent exploits the information of the complicated environment it resides for decision making, (iii) multiple parameters sensing and interpretation, (iv) tasks execution.

The objective of each agent was to accomplish a task assigned, taking into account rules or limitations, e.g., a deadline. In order to obtain its goals, it initially gathered data from its environment and consequently developed knowledge and/or makes use of relevant data or knowledge of its neighboring agents. Data, knowledge, and previous experience fuel a reasoning module, usually implemented with machine learning methods [2,3] that performs decision making. This concept allows for the wide application of agents in even complicated problems.

A special category of intelligent agents are the mobile ones (Mobile Agents—MAs). They consist of 3 major components: Code, state, and features. The code is the software that defines its behavior. The state is responsible for controlling a MA and managing the variables and objects stored within it. The features include: Autonomy, interactivity, adaptability, mobility, collaboration, intelligence, coordination, learning [4].

Internet of Things (IoT) is one of the most significant technologies that is expected to dominate the Internet. It comprises a network of devices, software entities, and agents that have the potential to communicate with each other, allowing for sharing and common storage of information without any human intervention. The most popular IoT devices are sensors that are widely applied in areas varying from industrial automation and environmental monitoring to military defense and agriculture. However, the healthcare domain emerges as one of the most popular scopes of application of such sensors (e.g., transparent data collection and transmission, minimal size) as patients’ continuous monitoring is a critical requirement for a HIT system.

#### 2.1.1. Significant Applications of Mobile Agents for Remote Medical Services

Mobile agents provide an inherent way of tackling distributed problems of accessing heterogeneous and spatially diverse data sources due to their cooperation and coordination abilities [5]. The applications of mobile and intelligent agents in healthcare have already been acknowledged in a previous literature survey conducted by Bagga and Hans [6]. They vary and range from medical data manipulation, information retrieval, and patient management to resource planning and remote medical support. As laid down from the literature, the main contributions of applying mobile agents in the healthcare domain were:

- Remote medical support and enhanced healthcare services.
- Information retrieval and integration: Acting as brokers between systems dedicated to retrieve, analyze, and protect essential medical information.
- Patients and resource planning: Coordinate and plan of the healthcare center resources.

The following sections review several studies that describe the most significant applications referred to in the literature and focus on the identified domains.

#### 2.1.2. Remote Medical Support and Monitoring

Chuan-Jun Su et al. [7] developed a MAS for data collection from women during pregnancy. Monitoring a human embryo allows for data collection and transmission, posing no limitations to the women’s mobility. Scalability and interoperability issues were manipulated by adopting the FIPA2000 standard.

Aminian M. et al. [8] presented a vital signals monitoring system for multiple patients. The system coordinator (e.g., a physician) communicates with the sensors of each patient in order to collect measurements. It can detect potential abnormal measurements and alert not only the patient but also inform her doctor through SMS or email. In addition, appropriate network topologies allow for minimal energy consumption that prolongs the network’s life span.

The idea of interconnecting physicians with patients anywhere, anytime, and over heterogeneous devices was proposed by Bouchemal et al. [9]. The system was implemented over the Java Agent development environment with Light weight extensible Agent platform—JADE-LEAP platform and deployed the Ubiquitous Mobile Agent (UMA), the Device Agents (DevA), and the Patient Agents (PatA) through the cloud, in order to alert the physician or to receive health status information on its behalf.

Toader [10] addressed the issue of combining IoT solutions and multi-mobile and intelligent agents that process medical data and make decisions. A top-down architecture was proposed that included not only a prototype software application but also an e-health platform that was developed over the Raspberry-Pi hardware module.

Z. Chaouch and M. Tamali [11] proposed a relevant system for the case of diabetic patients. Its primary objective was real-time monitoring and continuous healthcare provision in order to reduce incurring costs and patients and/or healthcare professionals commuting.

Research performed by W. Hsu and J. Pan [12] focused on designing a MAS based Peer-to-Peer (P2P) solution for remote and secure provision of medical services. Furthermore, this architecture allowed for interoperability among healthcare units in order to perform telesurgeries, elearning, and tele-consulting. In case of massive destructions, agents take care of coordinating the hospitals and provide all the necessary information to the stakeholders.

An agent-based solution, called vhMentor, that overcomes the automation deficiencies of medical data monitoring was proposed in [13]. The authors studied the applicability and usefulness of the mobile agent technology in the healthcare domain by encapsulating remote medical device signals in a solid ontological schema.

A smart ambulance service was being proposed by Alami-Kamour et al. [14] that takes advantage of a mobile agent platform thus as to allow nurses to give first aid to patients while being transferred to a hospital.

#### 2.1.3. Information Management, Integration, and Resource Planning

The technology of MAs has been acknowledged as a promising area of research for: The development of value-added information systems [15]. Collaboration is one of their intrinsic features thus as to form a dialogical framework for optimal service delivery and data collection. Different theoretical development aspects are usually introduced that include not only object-oriented programming but also artificial intelligence methods.

An energy-efficient end-to-end solution was proposed by Thangaraj et al. [16] that enabled the semantic communication of IoT medical devices. The resulting research proposal attempted to: (i) Improve the energy efficiency of the IoT devices that lack physical resources, (ii) boost data accuracy, (iii) deliver knowledge through ontology mapping, and (iv) perform risk factor prediction. A mobile health analytics platform was presented in detail by Ghosh et al. [17]. The platform was able to keep track and analyze biological signals that allow for the personalized health services provision to patients suffering from hypertension. Through a sophisticated dashboard, it is anticipated that the system will engage both patients and doctors in an efficient and effective primary care process.

Chen et al. [18] proposed a stable and scalable system based on mobile agents for secure access and retrieval mechanisms on patients’ data thus as to provide universal access, instant support, personalization, and improvement of Quality of Living. The security mechanism was based upon the CHINESE Remainder Theorem, and data retrieval included institutions and patients’ homes. C.H. Liu et al. [19] elaborated on the interoperability of Medical Information Systems that were spatially dispersed through mobile agents. Mobile Agents assisted the transmission and integration of data to the participating healthcare units. Consequently, patient data were provided in real-time and save time, unnecessary and recurring medical tests were avoided, and it saves medical resources.

A research project by Pouyan et al. [20] implemented a three layers agent model and supported the provision of healthcare services either remotely or on-site. The agents formed a dynamic virtual team consisting of medical professionals, nurses, and other stakeholders. Each patient was assigned to a medical group by an agent that undertakes patients’ data management.

A healthcare application developed by [21] proposed an approach that, apart from allowing the patient unhindered monitoring, established a secure framework over a blockchain infrastructure. OptiPres [22] is a mobile agent system designed to support the process of optimal drug prescribing and subjective decision-making under multiple parameters. The decision-making process was implemented as embedded behavior in the system’s agents.

### 2.2. An Overview of Healthcare Data Manipulation in the Digital Era

As the healthcare sector shifts in the digital era, increased volumes of data are being produced from various sources that include medical equipment and tools, research findings, and electronic health records, to name a few. These sources are producing data in various formats while their integration is becoming more and more necessary, mainly due to the decentralized nature of the applications and the interoperability requirements posed by the industry and interdisciplinary research efforts. Within this new ecosystem, relational databases cannot meet the availability and scalability requirements posed by web and mobile applications. Furthermore, big data analytics seem to be oppressed by the strict rules and limitations of the relational data model. As the unstructured data keep growing due to their sources’ diverse nature, the solution that gains attention among the research community and the industry is the NoSQL architecture that fits properly in the increasing performance and volume requirements of the database tier.

Numerous research efforts have adopted the NoSQL paradigm in order to design and implement scalable and flexible applications that can manipulate big data sources even in real-time. A combination of relational and non-relational databases is being proposed as a Clinical Data Repository by Hak et al. [23], thus as to refrain from the inefficiencies of traditional database systems. The proposed architecture exploits the characteristic of horizontal scalability to build distinct Storage Nodes within Zones that represent physical locations.

The potential of the NoSQL architecture appears so attractive that several articles attempted to evaluate the performance of NoSQL databases in the scope of the healthcare field [24,25]. Big data analytics applications that lend support to the claim that innovative information and computer technologies will boost research progress in the healthcare domain. NoSQL databases have gathered the attention of research efforts as they are able to address the challenges posed. To this end, a prototype architecture was proposed, named Med-BDA, that made use of state-of-the-art technologies to propose a roadmap for the adoption of Big Data analytics in the healthcare domain [26].

A NoSQL model [27] for storing health data was implemented in the Cloud for distribution purposes. The model’s efficiency was compared against the RDBMS model in terms of querying time, data preparation, flexibility, and extensibility criteria. As an overall assessment, the proposed model was found to surpass the RDBMS one in performance.

The evolution and necessity of BDA technologies seem so inevitable a platform was implemented for the Czech Republic’s healthcare system [28]. The horizontal scalability tests performed demonstrated a 25% performance improvement, and the system has already been employed in the battle against the coronavirus as a visualization middleware and a data exchange node with other healthcare information systems. According to Almeida et al. [29], one of the hottest research tasks in health data manipulation is that of accessing clinical data over distributed databases. They proposed two solutions to the problem: The first one required the deployment of a common data model that seemed rather optimistic; the second one, the semantic web approach, employed SPARQL queries over data that were converted into RDF format. Each method has its pros and cons; the common data model performs better in terms of cost, efficiency, and consistency, while the semantic web one surpasses the common data model in the interoperability and extendibility criteria.

As the need for big data management is constantly increasing, new tools and techniques are being developed. The Apache Hadoop ecosystem is dominating the field, and the various tools offered within are thoroughly presented in [30,31]. A NoSQL clinical data repository is described in [32] that provides convenient access to data, advanced analytics, and seamless integration. Its objective is to combine data from multiple data sources and make them available for exploration and decision making through a solution that is flexible due to the NoSQL Apache HBase database and robust enough as it includes the Apache Phoenix relational query engine.

### 2.3. Literature Review Findings

A common factor of all the above-mentioned studies was the deployment of mobile agents and unstructured data databases for tasks coordination and information integration from heterogeneous sources on behalf of a user.

According to the literature review findings, we identified three areas of MASs application in healthcare. In “remote medical support and healthcare services provision,” the systems described focused on taking the appropriate actions for patient support or providing digital healthcare services that could make a positive contribution to the treatment of chronic disease. The most common solution suggested in the articles studied was the one that every hardware module has its own software—mobile agent counterpart.

In the field of “information management, integration, and resource planning” innovative methods of data gathering and aggregation over heterogeneous devices of limited resources were being proposed. Thus, attention was attracted not only to the design of platform neutral techniques for data dissemination, while some of them also studied the problem of energy efficiency. An issue that has to be investigated in the future is that of cost-effectiveness analysis, as only a few of the systems proposed to take a step further by addressing it.

NoSQL technology was applied to tackle the problem of accessing and efficiently retrieving clinical data over distributed databases. Mainly, open-source frameworks were acknowledged in our survey for repository development, while the security issues that may arise were not specifically addressed.

The integration of dispersed info can lead to improved decision-making during healthcare provision. However, such systems are preliminary pilot studies, and further research efforts have to be put in order to be widely adopted as a standard solution by the industry. Stakeholders have to be involved during the design and development of such technical-intensive systems as the special nature of healthcare management systems is acknowledged (e.g., user interface design, requirements analysis, application programming interfaces, etc.).

## 3. Results

This section is devoted to describing an experimental system implemented over mobile agents for information retrieval in NoSQL environments. Its objective is to study the feasibility of developing systems that will employ ontological data representation and task implementation through mobile agents towards an innovative and flexible system for health data monitoring.

NoSQL technology has offered: The transparency of the design process and development of scalable applications, data aggregation with non-static and open schema from complex and diverse data sources, and the efficiency of the data management process in terms of throughput and latency.

Raw medical data were created for test and simulation purposes over an xml template, and the values assigned were medically correct. NoSQL databases were employed for storing the data. The choice of this technology was based on the fact that the medical data growth rate was increasing as sensor and IoT devices were more and more employed in healthcare provision. Furthermore, dynamic schemas for unstructured data whereas are most welcome against a well-designed pre-defined schema that SQL databases impose.

### 3.1. A Mobile Agent System for Information Retrieval in NoSQL Environments

**Figure 1:**
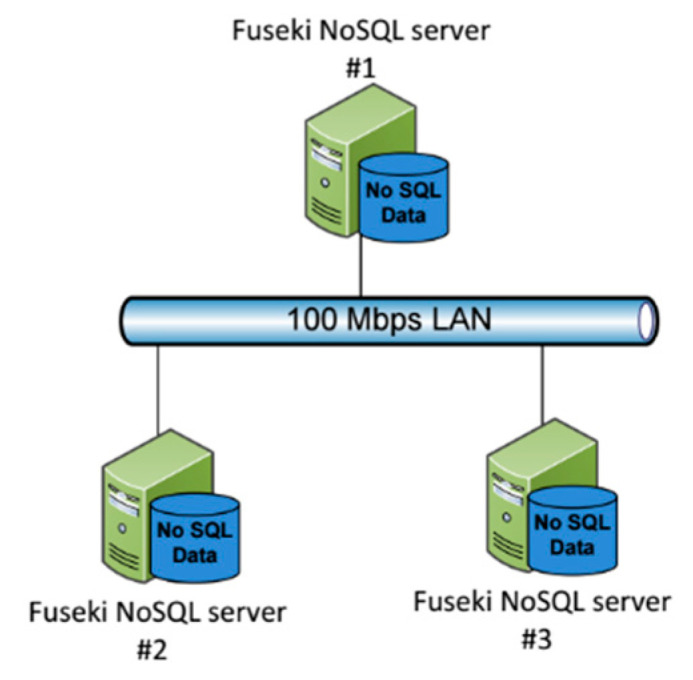
Fuseki NoSQL server installation.

**Figure 2:**
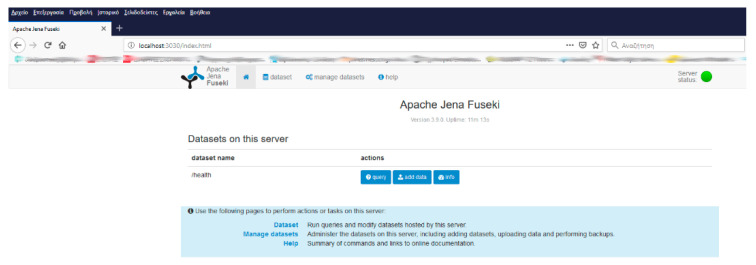
The “health” NoSQL database.

**Figure 3:**
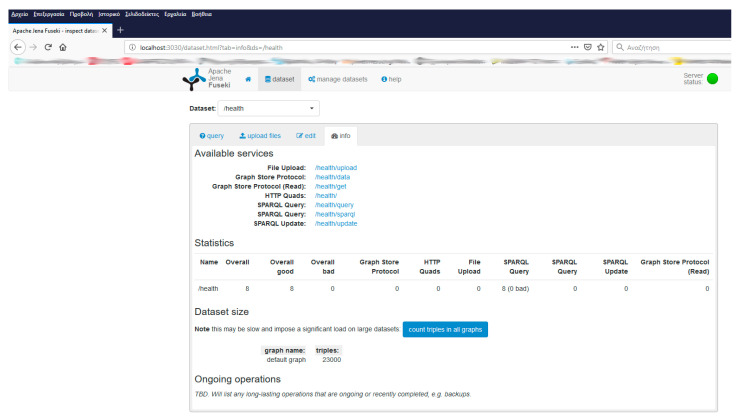
An overview of the database.

The experimental environment was based upon the following tools: Apache Jena Fuseki NoSQL server installed in three different hosts over a 100 Mbps LAN thus as to simulate NoSQL databases management as depicted in Figure 1. Figure 2 depicts the home page of Fuseki NoSQL server. For the requirements of the experimental setup, we deployed a database under the name “health” at each one of the three installations. The databases were populated with the demo raw data described above. The graph consisted of 23,000 triplets as shown in Figure 3.

A demo query was designed to retrieve the: Name, surname, health status, and diastolic pressure features for each patient. Subsequently, we implemented a distributed application based on mobile agents over the JADE platform.

JADE Mobile Agent Platform [33] is a middleware for the deployment of agent -based systems and implements the Foundation for Intelligent Physical Agents (FIPA) (The Foundation for Intelligent Physical Agents (FIPA), http://www.fipa.org/ (accessed on 2 February 2021)) specifications for interoperability of heterogeneous agents and services. It consists of the runtime environment, a complete agent API, and a set of graphical agent management tools. In JADE, the agent hosts are called Containers that is an instance of the JADE runtime environment. The following figure depicts the employed JADE Platform architecture, which includes a JADE platform that comprises of a Main-Container (A), with two simple Containers (1 and 2) registered on it. In such an environment, the agents are defined by their name and are able to communicate regardless of their actual location. The Main Container, apart from the agents it may host, is also the place that the Agent Management Service (AMS) and Directory Facilitator (DF) reside. AMS is a special type of agent that provides naming services to the platform while it enforces authority services. DF is a Yellow Pages agent and maintains a catalog of services provided by other agents within the platform. It is also worth mentioning that JADE is an independent operating system and requires minimal resources for its execution on Java-enabled devices.

**Figure 4:**
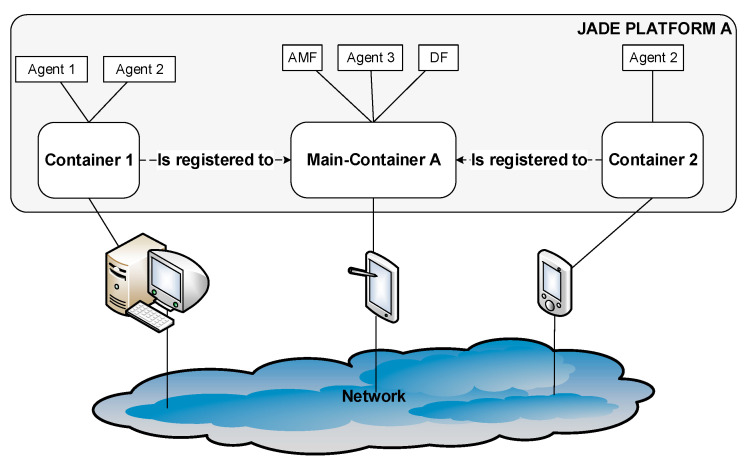
The JADE-based Platform architecture.

**Figure 5:**
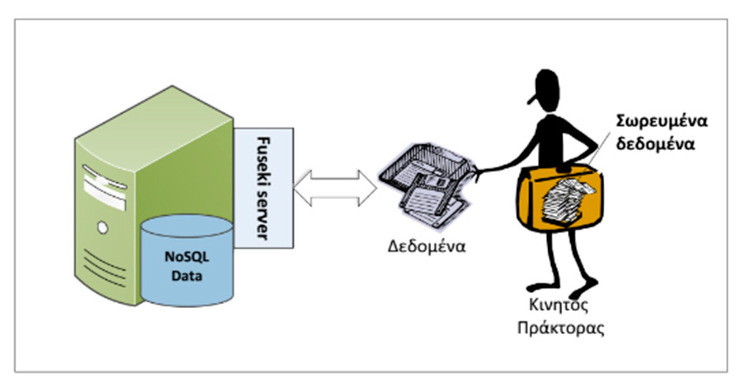
Mobile agent (MA) and Fuseki server interaction.

The containers initiated for the experimental setup were named Main Container, Container-1, and Container-2 (see Figure 4). The mobile agent is in explicit and continuous interaction with the NoSQL Fuseki Server (as described in Figure 5) and is responsible for issuing NoSQL queries, collecting the results, and storing them during its trajectory within the experimental environment. In addition, it preprocesses and aggregates the data in order to minimize resource consumption (storage, cpu, memory, and bandwidth).

**Figure 6:**
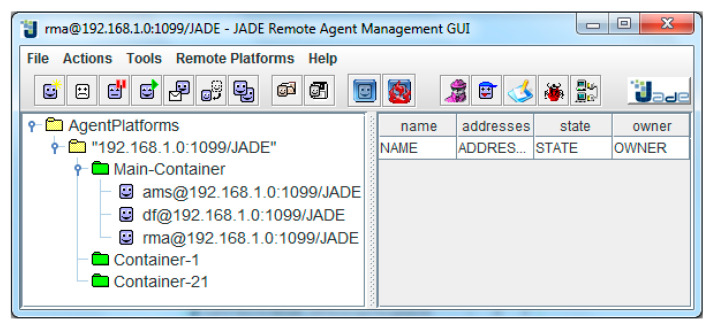
The GUI of the Main Container.

Figure 6 depicts the GUI of the Main Container where Container-1 and Container-2 are already initialized and interconnected. Thereafter the system is ready for initializing the mobile agent.

### 3.2. Experimental Results

The configuration chosen for this experiment deployed only one mobile agent. However, for load balancing reasons and efficiency improvement, there were several studies proposing the deployment of additional agents allowing for the segmentation of a network topology in clusters according to features that may include: The node processing load, available energy for energy-autonomous devices, available bandwidth, etc. Each mobile agent was assigned a cluster for monitoring and data collection. The complexity posed by such an architecture was eliminated by the communication capabilities among the mobile agents.

**Figure 7:**
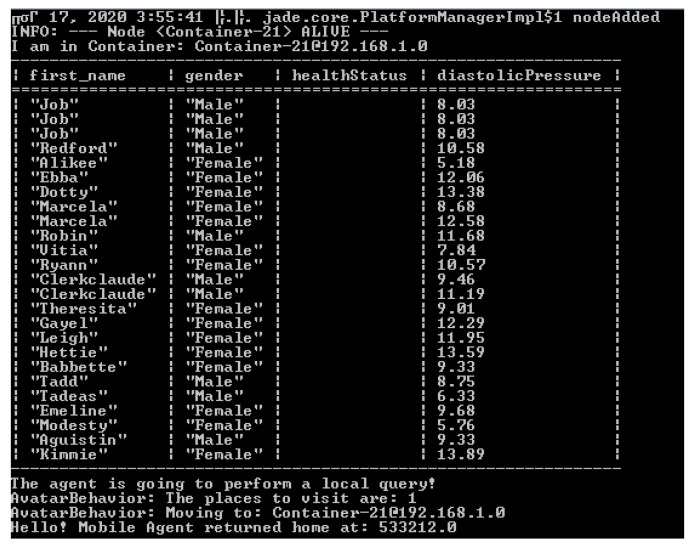
The result of a mobile agent’s tasks execution in the Main container.

In order to benchmark the proposed architecture, a variant system was implemented based on a relational database. The query was the same in both versions. Figure 7 depicts the comparison results where 10 consecutive executions of each architecture were performed thus as to avoid any resource overloading bias. The results revealed that the NoSQL implementation outperformed the standard relational one as the calculated average percentage difference of the execution time was 90.77%.

**Figure 8:**
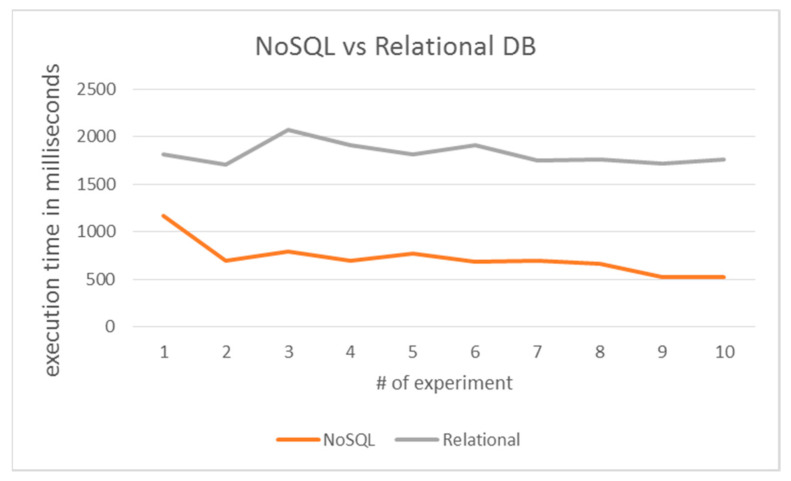
Comparison of NoSQL vs. Relational database query execution.

A reason that contributes to the increased execution time of the relational implementation was that the JDBC connection needs considerable time to establish and measurements revealed that it takes more than 900 ms (Figure 8).

## 4. Conclusions

The requirements of healthcare services are inherently distributed and complex. Thus, MAs along with NoSQL databases appear to be a promising and effective solution in the field that provides a services and data abstraction layer and not a concrete reference technology. Such an approach is instantiated ad-hoc according to the available technologies and the requirements of the system under development. As a healthcare information management ecosystem gathers all these features, the holistic data and services management demands for common and distributed processes of decision making through communication and cooperation among systems, organizations, and humans.

However, the MAs-based healthcare solutions should face several challenges that need to be addressed and include: User expectations and user acceptance, the decentralization of management control, MA technology adoption from the industry, etc. Non-technical aspects also have to be confronted and span from law and ethical issues to privacy protection, data integrity, and authentication. Unfortunately, there is often a gap between research efforts in information technology and real requirements of a healthcare system that poses a burden in the wide adoption of MAs applications. Furthermore, most of the proposed solutions suffer a lack of testing data as well as a cost efficiency assessment (e.g., the ROI of the provided services is not well defined).

This work initially attempted a literature review of the applications based on MA technologies for healthcare support services, and it is encouraging to note the growing interest in this technology as a candidate solution to the intrinsic complexity and distributed nature of the problem. The articles included exploiting MASs as a solution for integrating vital signals sensing devices in order to support patients and provide healthcare services. In addition, attention is put on the design of platform neutral techniques for developing clinical data repositories over NoSQL technology.

Cost-effectiveness analysis of the proposed solutions constitutes an issue that has to be further investigated. Only a few of the systems proposed to take a step further by addressing it. This is largely due to the emerging nature of the technologies studied, and their adoption is limited in the healthcare domain.

Subsequently, we implemented a pilot system that merges the prominent technologies of the literature review. As a feasibility study, the system performed data retrieval tasks over demo NoSQL databases. The pilot was qualitatively investigated for its stability and could be effectively adopted for patients’ monitoring. Performance benchmarking experiments were conducted to assess the role of the data back-end (NoSQL vs. relational model). The results revealed the poor performance of the relational databases compared to the NoSQL ones. That was mainly down to the JDBC overhead of relational databases and the inherent lightweight design of NoSQL data model.

Future research plans will focus on the exhaustive assessment of the proposed system over real medical data sources. In addition, the system could be enriched with inference and data sources merging capabilities. Furthermore, efforts will be devoted to the evaluation of the users’ acceptance and satisfaction of such a data retrieval system. Ideally, the evaluation should be combined with a relevant medical application that will provide added value and a clear orientation to the test case.

It seems inevitable that along with the Millennials generation aging, we will witness increasing healthcare support services. As a result, the MA approach could be adopted and shape the healthcare field by providing appropriate solutions towards meeting the rise in demand for health care. More specifically, such solutions are expected to increase the possibility to “age at home” and avoid institutionalization, while developments in the field of IoT and health sensors are expected to enhance the MA applications in terms of usability and efficiency.

## Data Availability

Data sharing not applicable.

## References

[1] Alami-Kamouri S., Orhanou G., Elhajji S., Longo A., Zappatore M., Villari M., Rana O., Bruneo D., Ranjan R., Fazio M., Longo A. (2017). Mobile Agent Service Model for Smart Ambulance. Lecture Notes of the Institute for Computer Sciences, Social Informatics and Telecommunications Engineering.

[2] Almeida J., Fajarda O., Pereira A., Oliveira J. Strategies to Access Patient Clinical Data from Distributed Databases. Proceedings of the 12th International Joint Conference on Biomedical Engineering Systems and Technologies.

[3] Alruqi M., Hsairi L., Eshmawi A. Secure mobile agents for patient status telemonitoring using blockchain. Proceedings of the 18th International Conference on Advances in Mobile Computing & Multimedia.

[4] Aminian M., Naji H.R. (2013). A Hospital Healthcare Monitoring System Using Wireless Sensor Networks. J. Health Med. Inform..

[5] Bagga P., Hans R. (2015). Applications of Mobile Agents in Healthcare Domain: A Literature Survey. Int. J. Grid Distrib. Comput..

[6] Bosse S. Distributed Machine Learning with Self-Organizing Mobile Agents for Earthquake Monitoring. Proceedings of the 2016 IEEE 1st International Workshops on Foundations and Applications of Self* Systems (FAS*W).

[7] Bouchemal N., Maamri R., Bouchemal N. (2019). Telemonitoring Healthcare System-Based Mobile Agent Technology. Intelligent Systems for Healthcare Management and Delivery.

[8] Celesti A., Lay-Ekuakille A., Wan J., Fazio M., Celesti F., Romano A., Bramanti P., Villari M. (2020). Information management in IoT cloud-based tele-rehabilitation as a service for smart cities: Comparison of NoSQL approaches. Measurement.

[9] Chaouch Z., Tamali M. (2014). A Mobile Agent-Based Technique for Medical Monitoring (Supports of Patients with Diabetes). Int. J. Comput. Model. Algorithms Med..

[10] Chen T.-L., Chung Y.-F., Lin F.Y.S. (2011). Deployment of Secure Mobile Agents for Medical Information Systems. J. Med. Syst..

[11] Christopoulou S., Kotsilieris T., Anagnostopoulos I. A health care monitoring system that uses ontology agents. Proceedings of the 2016 11th International Workshop on Semantic and Social Media Adaptation and Personalization (SMAP).

[12] Franklin S., Graesser A. (1997). Is It an agent, or just a program?: A taxonomy for autonomous agents. Lecture Notes in Computer Science.

[13] Freire S.M., Teodoro U., Wei-Kleiner F., Sundvall E., Karlsson D., Lambrix P. (2016). Comparing the Performance of NoSQL Approaches for Managing Archetype-Based Electronic Health Record Data. PLoS ONE.

[14] Ghosh A., Stepanov E.A., Torres J.M.M., Danieli M., Riccardi G. HEAL: A Health Analytics Intelligent Agent Platform for the acquisition and analysis of physiological signals. Proceedings of the 2018 IEEE 20th International Conference on e-Health Networking, Applications and Services (Healthcom).

[15] Goli-Malekabadi Z., Sargolzaei-Javan M., Akbari-Fatidahi M.K. (2016). An effective model for store and retrieve big health data in cloud computing. Comput. Methods Programs Biomed..

[16] Hak F., Guimarães T., Abelha A., Santos M. (2020). An Exploratory Study of a NoSQL Database for a Clinical Data Repository. Advances in Intelligent Systems and Computing.

[17] Hsu W.-S., Pan J.-I. (2013). Secure Mobile Agent for Telemedicine Based on P2P Networks. J. Med. Syst..

[18] Imran S., Mahmood T., Morshed A., Sellis T. (2021). Big data analytics in healthcare A systematic literature review and roadmap for practical implementation. IEEE/CAA J. Autom. Sin..

[19] Karami M., Shahmirzadi A.H. (2018). Applying Agent-based Technologies in Complex Healthcare Environment. Iran. J. Public Health.

[20] Kumar S., Singh M. (2019). Big data analytics for healthcare industry: Impact, applications, and tools. Big Data Min. Anal..

[21] Liu C.-H., Chung Y.-F., Chiang T.-W., Chen T.-S., Wang S.-D. (2011). A Mobile Agent Approach for Secure Integrated Medical Information Systems. J. Med. Syst..

[22] Livieris I., Kanavos A., Pintelas P. (2019). Detecting Lung Abnormalities From X-rays Using an Improved SSL Algorithm. Electron. Notes Theor. Comput. Sci..

[23] Miller K., Mansingh G. (2017). OptiPres: A distributed mobile agent decision support system for optimal patient drug prescription. Inf. Syst. Front..

[24] Pintelas E., Liaskos M., Livieris I.E., Kotsiantis S., Pintelas P. (2020). Explainable Machine Learning Framework for Image Classification Problems: Case Study on Glioma Cancer Prediction. J. Imaging.

[25] Pouyan A.A., Ekrami S., Taban M. A distributed E-health model using Mobile Agents. Proceedings of the Seventh International Conference on Autonomic and Autonomous Systems.

[26] Shastri A., Deshpande M. (2019). A Review of Big Data and Its Applications in Healthcare and Public Sector. Big Data Anal. Healthc..

[27] Štufi M., Bačić B., Stoimenov L. (2020). Big Data Analytics and Processing Platform in Czech Republic Healthcare. Appl. Sci..

[28] Su C.-J., Chu T.-W. (2014). A Mobile Multi-Agent Information System for Ubiquitous Fetal Monitoring. Int. J. Environ. Res. Public Health.

[29] Telecom Italia Lab JAVA Agent DEvelopment Framework. http://jade.tilab.com.

[30] Thangaraj M., Ponmalar P.P., Sujatha G., Anuradha S. Agent based Semantic Internet of Things (IoT) in Smart Health care. Proceedings of the 11th International Knowledge Management in Organizations Conference on The Changing Face of Knowledge Management Impacting Society.

[31] Toader C.G. Multi-Agent Based E-Health System. Proceedings of the 2017 21st International Conference on Control Systems and Computer Science (CSCS).

[32] World Health Organization (2018). Ageing and Health. https://www.who.int/news-room/fact-sheets/detail/ageing-and-health.

[33] Yang E., Scheff J.D., Shen S.C., A Farnum M., Sefton J., Lobanov V.S., Agrafiotis D.K. (2019). A late-binding, distributed, NoSQL warehouse for integrating patient data from clinical trials. Database.

